# RNA Exosome Component EXOSC4 Amplified in Multiple Cancer Types Is Required for the Cancer Cell Survival

**DOI:** 10.3390/ijms23010496

**Published:** 2022-01-02

**Authors:** Kenzui Taniue, Tanzina Tanu, Yuki Shimoura, Shuhei Mitsutomi, Han Han, Rika Kakisaka, Yusuke Ono, Nobue Tamamura, Kenji Takahashi, Youichiro Wada, Yusuke Mizukami, Nobuyoshi Akimitsu

**Affiliations:** 1 Isotope Science Center, The University of Tokyo, Tokyo 113-0032, Japan; tanu@ric.u-tokyo.ac.jp (T.T.); shimoura@ric.u-tokyo.ac.jp (Y.S.); shuheimitsutomi@gmail.com (S.M.); han@ric.u-tokyo.ac.jp (H.H.); wada-y@lsbm.org (Y.W.); 2 Cancer Genomics and Precision Medicine, Department of Medicine, Asahikawa Medical University, Asahikawa 078-8510, Japan; ntamamura@asahikawa-med.ac.jp (N.T.); t-kenji@asahikawa-med.ac.jp (K.T.); mizu@asahikawa-med.ac.jp (Y.M.); 3 Institute of Biomedical Research, Sapporo Higashi Tokushukai Hospital, Sapporo 065-0033, Japan; kakisaka.rika@higashi-tokushukai.or.jp (R.K.); y-ono_ccbr@tohtoku.jp (Y.O.)

**Keywords:** TCGA, PanCancer, RNA exosome, gene amplification, EXOSC4, pancreatic cancer, cell survival

## Abstract

The RNA exosome is a multi-subunit ribonuclease complex that is evolutionally conserved and the major cellular machinery for the surveillance, processing, degradation, and turnover of diverse RNAs essential for cell viability. Here we performed integrated genomic and clinicopathological analyses of 27 RNA exosome components across 32 tumor types using The Cancer Genome Atlas PanCancer Atlas Studies’ datasets. We discovered that the *EXOSC4* gene, which encodes a barrel component of the RNA exosome, was amplified across multiple cancer types. We further found that *EXOSC4* alteration is associated with a poor prognosis of pancreatic cancer patients. Moreover, we demonstrated that EXOSC4 is required for the survival of pancreatic cancer cells. EXOSC4 also repressed *BIK* expression and destabilized *SESN2* mRNA by promoting its degradation. Furthermore, knockdown of BIK and SESN2 could partially rescue pancreatic cells from the reduction in cell viability caused by EXOSC4 knockdown. Our study provides evidence for EXOSC4-mediated regulation of *BIK* and *SESN2* mRNA in the survival of pancreatic tumor cells.

## 1. Introduction

RNA decay plays a crucial role in the post-transcriptional regulation of gene expression [1]. The human RNA exosome is a highly conserved ribonuclease complex that is critical for both the processing and degradation of various kinds of RNAs [2]. The RNA exosome is composed of a catalytically inactive barrel structure of nine core subunits (known as EXO9) that achieves its catalytic activity via the interaction with the exoribonuclease exosome component 10 (EXOSC10), the exo/endo-ribonuclease DIS3, and the two DIS3-like proteins (the exoribonucleases DIS3L and DIS3L2) [2,3,4,5,6,7]. Accessory protein C1D directly forms a stable heterodimer with EXOSC10 and facilitates its function, whereas the other accessory protein MPHOSPH6 also associates with the EXO9 but with a less established molecular function [2,5,8]. The DiGeorge critical region 8 (DGCR8), a double-stranded RNA-binding protein involved in miRNA biogenesis, acts as an adaptor protein to recruit the exosome to mature snoRNAs and human telomerase RNA for degradations [8,9]. In addition, nuclear exosome function critically relies on the DExH/D box RNA helicase, MTR4, which serves to direct substrates into the exosome barrel as well as to provide a platform for recruiting additional exosome adaptor proteins, specifying nuclear RNA targets [5,8,10]. In human cells, several adaptor complexes exist, but three are especially well described. The Trf4p/Air2p/Mtr4p polyadenylation (TRAMP) complex localizes to nucleoli and is predominantly involved in exosomal rRNA decay [11,12,13,14]. Conversely, the nuclear exosome-targeting (NEXT) complex and the pA tail exosome-targeting (PAXT) connection are localized in the nucleoplasm [12,15]. Furthermore, in our previous study, we found that hnRNPH1 interacts with MTR4 and is required for the MTR4-dependent turnover of lncRNA *NEAT1v2* [16]. On the other hand, the superkiller (Ski) complex is necessary for mRNA turnover, degradation of aberrant mRNAs, viral defense, and RNAi machineries in the cytoplasm [17,18]. Moreover, the short splicing isoform of human HBS1L (HBS1Lv3) bridges the Ski complex and RNA exosome [17,18,19,20].

The RNA exosome complex is localized in both the nucleus and the cytoplasm, with changing compositions and activities [21]. Recently, mutations in genes encoding RNA exosome subunits have been linked to several human diseases, including neurodegenerative diseases, retinitis pigmentosa, mild intellectual disability, and multiple myeloma [18,22,23,24,25,26,27]. Several groups have already reported that RNA exosome components regulate cell proliferation and apoptosis. It has recently been reported that DIS3 depletion induced apoptosis in erythroid precursor cells [28] and knockdown of EXOSC8 and EXOSC9 in human colon cancer cells leads to G2/M cell cycle arrest [29]. It has also been shown that EXOSC3 depletion leads to the increase in cleavage caspase 3 signal and the genomic instability via accumulation of R-loop structures [30,31]. However, the role of RNA exosome components in cancer progression is still not well known.

**Figure 1:**
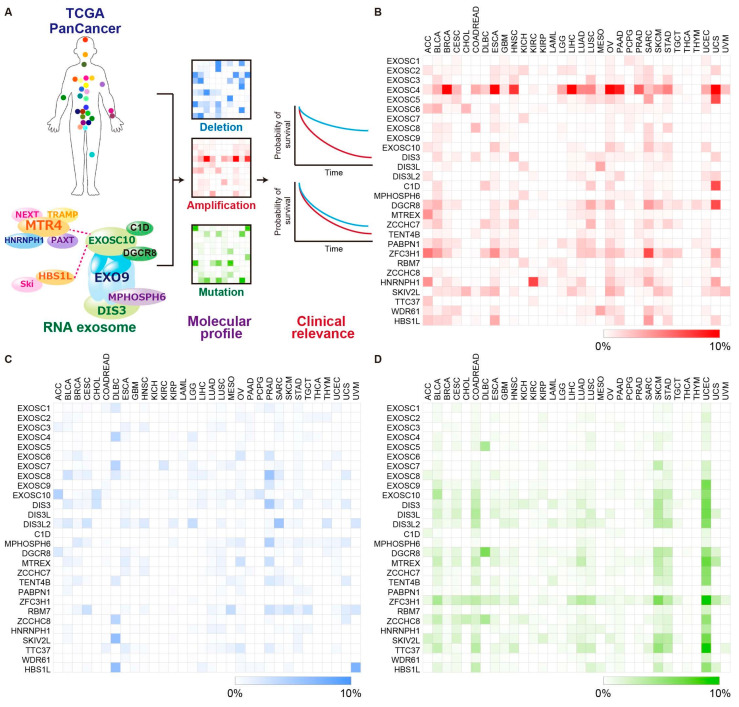
Molecular profiling of RNA exosome components in human cancer. (**A**) Schematic of the strategy to identify potential RNA exosome components that have important roles in cancer progression. (**B**–**D**) Heatmaps showing the frequencies of RNA exosome component amplification (**B**; red), deep deletion (**C**; blue), and mutation (**D**; green) across all 32 TCGA tumor types.

In the present study, we hypothesized that RNA exosome components with recurrent genetic alterations may play critical roles in cancer progression and could be novel therapeutic targets for cancer treatment (Figure 1A). We found that the gene encoding exosome component 4 (EXOSC4), an EXO9 component, was amplified in multiple cancer types using the data from The Cancer Genome Atlas (TCGA) PanCancer Atlas. We also found that *EXOSC4* alteration was associated with poor disease prognosis in pancreatic cancer. We then focused on EXOSC4 in pancreatic cancer and investigated its role in pancreatic cancer cell viability and found that EXOSC4 knockdown led to the reduction in the growth and increase in the apoptotic cell death of pancreatic cancer cells. In addition, GO and GSEA analyses revealed that the genes regulated by EXOSC4 in pancreatic cancer cells were enriched for genes involved in lysosome and apoptosis. Furthermore, EXOSC4 knockdown increased mRNA levels of *BIK* and *SESN2*, which regulate apoptosis in pancreatic cancer cells. Altogether, our results suggested that EXOSC4 inhibition may be a novel therapeutic approach for pancreatic cancer treatment.

## 2. Results

### 2.1. EXOSC4 Is Amplified and Upregulated in Pancreatic Cancer Tissue

The human genome contains 27 genes that encode RNA exosome components (Table S1) [5,8]. To identify their genomic alterations in human cancer, we examined their copy number alterations (CNA) and mutation profiles in tumor samples across 32 tumor types from TCGA PanCancer Atlas Studies (Table S2) [32,33]. The amplification, deep deletion, and mutation data of 10,967 samples from the TCGA Pan-Cancer Atlas were obtained from cBioPortal [34]. CNAs of RNA exosome components were derived from the Genomic Identification of Significant Targets in Cancer (GISTIC) algorithm. TCGA PanCancer analysis revealed that *EXOSC4* showed high-level amplification above 5% in nine different cancer types, including Breast invasive carcinoma (BRCA), Esophageal carcinoma (ESCA), Head and Neck squamous cell carcinoma (HNSC), Liver hepatocellular carcinoma (LIHC), Ovarian serous cystadenocarcinoma (OV), Pancreatic adenocarcinoma (PAAD), Prostate adenocarcinoma (PRAD), Stomach adenocarcinoma (STAD), and Uterine Carcinosarcoma (UCS) (Figure 1B and Figure S1A, and Tables S2 and S3). In addition, ZFC3H1 was amplified at a high level above 5% in only two cancer types, ACC and SARC (Figure 1B and Tables S2 and S3). No genes showed deep deletions or somatic mutations above 5% in more than three different cancer types in TCGA Pan-Cancer cohort (Figure 1C,D, and Tables S4 and S5). The *EXOSC4* gene is located on chromosome 8q24.3. Consistent with our results, array-Comparative Genomic Hybridization (CGH) and microarray analysis from several groups revealed that 8q24.3 was amplified in primary and metastatic tumors [35,36,37]. Next, we examined the co-amplification and co-expression of EXOSC4 with HSF1, MYC, and POU5F1B, which are located on chromosomes 8q24.3, 8q24.21, and 8q24.21, respectively, in nine different cancer types. We found that HSF1 was co-amplified and co-expressed with EXOSC4 in all nine cancer types (Figure S1B,C, and Tables S6 and S7). Meanwhile, we found that MYC and POU5F1B were co-amplified with EXOSC4 at a high frequency but co-expressed with low correlation in all nine cancer types (Figure S1B,C, and Tables S6 and S7).

**Figure 2:**
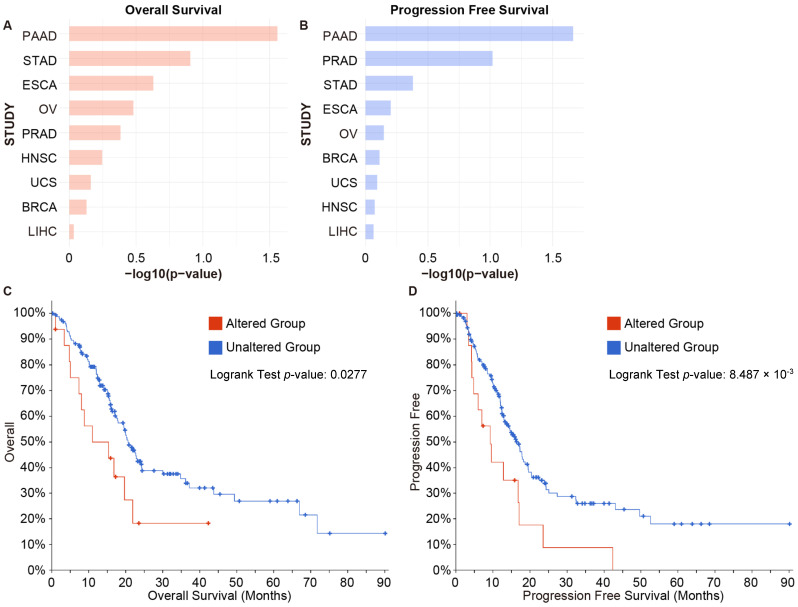
*EXOSC4* alteration is associated with poor survival and progression in the PAAD cohort. (**A**,**B**) The *p*-values for the Kaplan–Meier overall survival (**A**) and progression–free survival (**B**) curves for *EXOSC4* alteration in multiple cancer types. (**C**,**D**) Kaplan–Meier plots correlating *EXOSC4* alterations with time to overall survival (**C**) or to progression-free survival (**D**) in the case of pancreatic cancer.

To investigate the correlation of *EXOSC4* alteration with the outcome of cancer patients, we next examined overall and progression-free survival by Kaplan–Meier analysis using the cBioPortal database. A significant association was found between the alteration of *EXOSC4* and overall and progression-free survival of pancreatic cancer patients. Although there was no association between *EXOSC4* alteration and clinical outcomes of the eight cancer types other than pancreatic cancer (Figure 2 and Figure S2A,B and Tables S8 and S9).

We then validated the gene amplification of the *EXOSC4* gene, but not that of genes encoding other RNA exosome components, in pancreatic cancer patients using data from Pancreatic Cancer [UT Southwestern (UTSW)] by the cBioPortal database [38]. (Figure S3A). We next examined the expression of *EXOSC4* in human tumorous and noncancerous pancreatic tissues using the GEPIA 2 database, a web tool based on the TCGA database, and another public data set (GSE 43795 [39]). We found that *EXOSC4* expression was higher in pancreatic tumors than in noncancerous tissues (Figure S3B,C). We next performed immunohistochemical staining for EXOSC4 on primary pancreatic cancer tissues and tumor-adjacent pancreatic tissues from pancreatic cancer patients. EXOSC4 expression was identified in the cytoplasm and nucleus of tumor cells, while few cells showed EXOSC4 expression in tumor-adjacent pancreatic tissues (Figure S3D). Consistent with these results, immunofluorescence revealed that EXOSC4 is localized in both cytoplasm and nucleus of pancreatic cancer cells (Figure S3E). Taken together, these results suggested that *EXOSC4* is amplified and/or upregulated in pancreatic cancer tissues.

### 2.2. EXOSC4 Knockdown Causes a Reduction in the Growth of Pancreatic Cancer Cells

**Figure 3:**
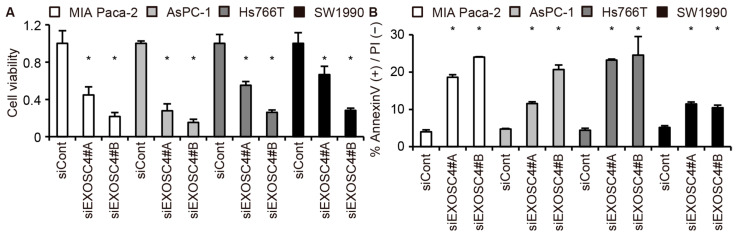
Knockdown of EXOSC4 reduces the growth of pancreatic cancer cells. (**A**) Viability of MIA Paca-2, AsPC-1, Hs766T, and SW1990 cells transfected with siRNA targeting EXOSC4 was assessed by Cell Counting Kit-8. Results are expressed as the mean ± s.d. (*n* = 4); * *p* < 0.05. (**B**) Annexin V assays were performed with MIA Paca-2, AsPC-1, Hs766T, and SW1990 cells transfected with the indicated siRNA. Results are expressed as the mean ± s.d. (*n* = 3); * *p* < 0.05.

To elucidate the role of the EXOSC4 in pancreatic cell tumorigenesis, we examined the impact of downregulating EXOSC4 by siRNA on cell viability. Cell proliferation assays revealed that knockdown of EXOSC4 by siRNA caused a marked reduction in the growth of the pancreatic cancer cell lines MIA Paca-2, AsPC-1, Hs766T, and SW1990 as well as the colorectal cancer cell line HCT116 cells (Figure 3A and Figure S4A–D). We also found that knockdown of EXOSC4 resulted in marked increases in the apoptotic death of MIA Paca-2, AsPC-1, Hs766T, and SW1990 cells, as determined by Annexin assays (Figure 3B and Figure S4A,B). We next examined whether other RNA exosome components regulate the proliferation of pancreatic cancer cells. We found that knockdown of EXOSC10, but not EXOSC3 or EXOSC9, caused growth inhibition of pancreatic cancer cells (Figure S4E,F). These results suggest that EXOSC4 is required for the survival of pancreatic tumor cells and not all RNA exosome components affect pancreatic cell proliferation.

### 2.3. EXOSC4 Downregulates BIK and SESN2 mRNAs in Pancreatic Cancer Cells

**Figure 4:**
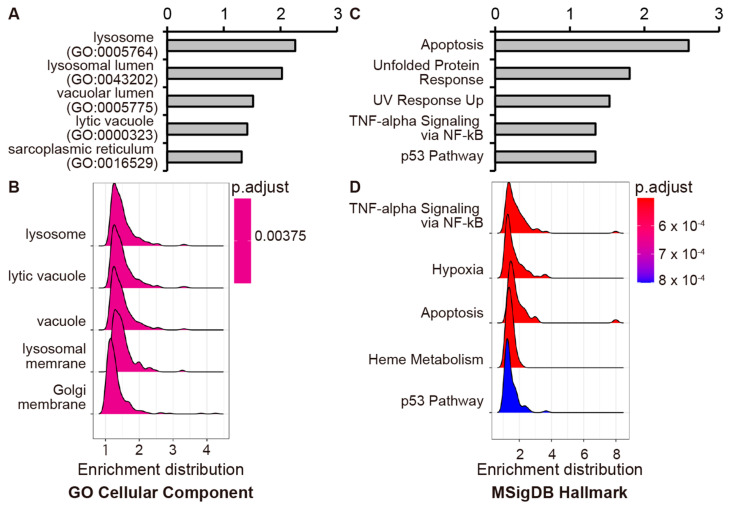
EXOSC4 regulates the genes involved in lysosome and apoptosis. (**A**) Enricher analysis of core EXOSC4-regulated genes using GO Cellular Component. (**B**) Ridgeplot showing GO Cellular Component category based on the GSEA analysis. (**C**) Enricher analysis of core EXOSC4-regulated genes using MSigDB Hallmark 2020 data set. (**D**) Ridgeplot showing MSigDB Hallmark category based on the GSEA analysis.

**Figure 5:**
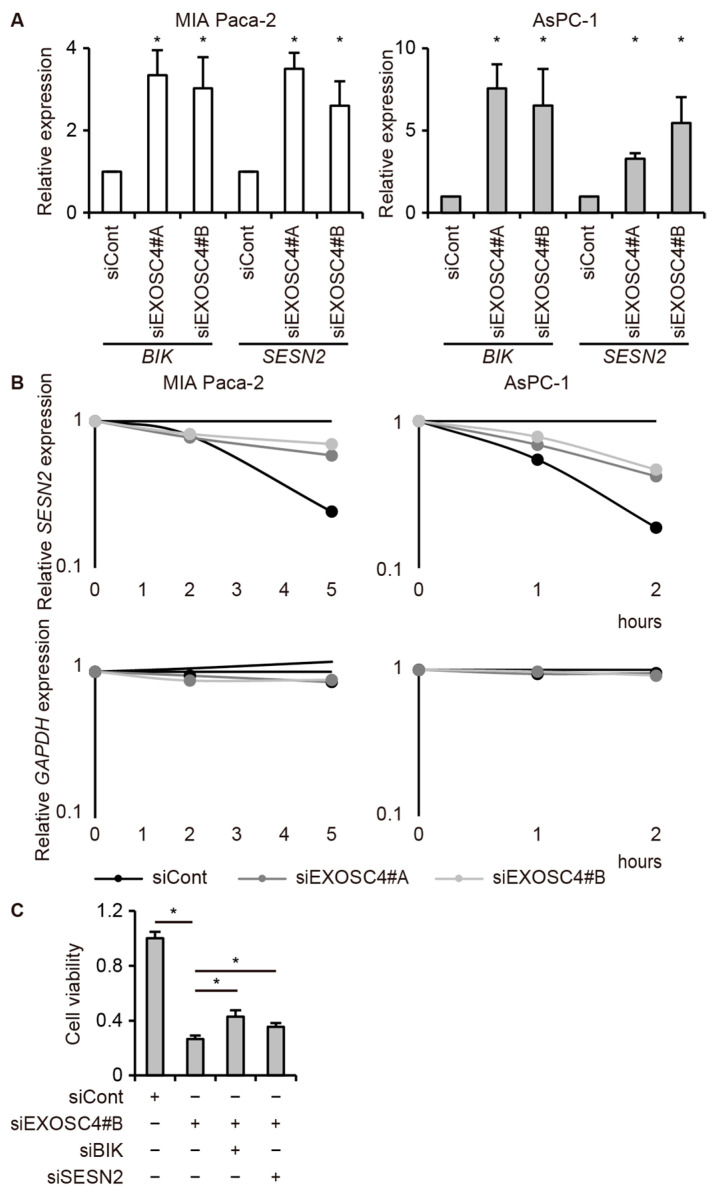
EXOSC4 regulates *BIK* mRNA expression and *SESN2* mRNA stabilization. (**A**) qRT-PCR analysis of *BIK* and *SESN2* expression in MIA Paca-2 (left) and AsPC-1 (right) cells transfected with siRNA targeting EXOSC4. Results are expressed as the mean ± s.d. (*n* = 3); * *p* < 0.05. (**B**) MIA Paca-2 (left) and AsPC-1 (right) cells transfected with siRNA targeting EXOSC4 were treated with 5, 6-dichloro-1-beta-D-ribofuranosyl benzimidazole for the indicated times and then subjected to qRT-PCR analysis. *GAPDH* mRNA was used as a negative control. In each degradation curve, data are shown as an exponential plot. (**C**) Viability of AsPC-1 cells transfected with an siRNA targeting EXOSC4 along with siBIK or siSESN2 was assessed by Cell Counting Kit-8. Results are expressed as the mean ± s.d. (*n* = 4); * *p* < 0.05.

To further elucidate the potential mechanism of EXOSC4 in pancreatic cancer cells, we analyzed EXOSC4-regulated genes by RNA-seq analysis and pathway analysis using Enrichr, a comprehensive gene set enrichment analysis web server [40,41,42]. Knockdown of EXOSC4 in MIA Paca-2 and AsPC-1 cells resulted in the up-regulation of 245 and 283 genes, respectively, as determined by RNA-seq analysis (Figure S5A, Tables S10–S12). We defined genes with upregulated expression in both EXOSC4 siRNA-transfected MIA Paca-2 and AsPC-1 cells as core EXOSC4-regulated genes (Figure S5A, Table S13). GO analysis by the Enrichr database revealed that the core EXOSC4-regulated genes were enriched for genes involved in lysosome (Figure 4A and Tables S13 and S14). Moreover, the ridgeplot and GSEAplot of the GSEA results also revealed that the genes involved in “lysosome” were activated in EXOSC-depleted cells (Figure 4B and Figure S5B and Tables S15 and S16). Consistent with our results above, Enrichr analysis revealed that the core EXOSC4-regulated genes were enriched for genes involved in apoptosis (Figure 4C and Tables S13 and S17). Moreover, GSEA analysis revealed that the genes involved in apoptosis were upregulated in EXOSC4 knockdown cells (Figure 4D and Figure S5C and Tables S15 and S18). We next found that BCL-2 interacting killer (*BIK*) and Sestrin2 (*SESN2*) mRNAs were upregulated in both MIA Paca-2 and AsPC-1 cells with EXOSC4 knockdown (Table S13). To validate the RNA-seq results, we performed qRT-PCR analysis and found that EXOSC4 knockdown led to the upregulation of *BIK* and *SESN2* mRNAs in both MIA Paca-2 and AsPC-1 cells (Figure 5A).

BIK is a pro-apoptotic BH3-only member of the BCL-2 family [43,44]. SESN2, a member of the SESN family, provides cytoprotection against various cellular processes, including reactive oxygen species’ production, DNA damage response, and cell viability [45,46,47]. A previous study reported that SESN2 induced apoptosis by regulating XIAP degradation [46]. To verify that EXOSC4 is involved in *BIK* and *SESN2* mRNA degradation, we measured turnover rates of these mRNAs in EXOSC4-depleted cells. The qRT-PCR analysis revealed that EXOSC4 knockdown increased the half-life of *SESN2* mRNA (Figure 5B). Endogenous *BIK* mRNA expression was already low and it was not possible to detect further effects on half-life. We concluded that EXSOC4 downregulates *BIK* mRNA and destabilizes *SESN2* mRNA in pancreatic cancer cells. Furthermore, we found that knockdown of BIK or SESN2 could partially rescue AsPC-1 cells from the reduction in cell viability caused by EXOSC4 knockdown (Figure 5C and Figure S6). These results suggest that EXOSC4 depletion causes BIK upregulation and *SESN2* mRNA stabilization and influences cell proliferation in pancreatic cancer cells.

## 3. Discussion

In this study, we examined RNA exosome components that may be critical for cancer progression. We performed integrated genomic and clinicopathological analyses of 27 RNA exosome components using the data across 32 cancer types from TCGA PanCancer Atlas, a large cohort of primary tumors. We found that the *EXOSC4* gene is amplified in multiple cancer types and its amplification was associated with poor disease prognosis in pancreatic cancer patients. Moreover, we found that EXOSC4 knockdown leads to the reduction in the growth of pancreatic cancer cells. We also found that GO and GSEA analyses revealed that EXOSC4 regulates genes encoding proteins that are involved in lysosome and apoptosis. Furthermore, we revealed that EXOSC4 knockdown induces *BIK* and *SESN2* mRNA levels in pancreatic cancer cells.

EXOSC4 is a barrel component of the RNA exosome [4]. A recent report revealed that EXOSC4 interacts with and is regulated by STX2 and promotes the proliferation of colorectal cancer cells [48]. In addition, EXOSC4 overexpression leads to the increase of viability, foci formation, invasiveness, and migration of normal and cancer colon cells [37] and its depletion decreases the proliferation of breast cancer cells [49]. Consistent with their results, we also showed that EXOSC4 knockdown led to the reduction in the viability of pancreatic and colorectal tumor cells, even though EXOSC4 alteration is associated only with a poor prognosis of pancreatic cancer patients. In addition, we found that EXOSC4 knockdown caused the upregulation in mRNAs’ levels of *BIK* and *SESN2*, both of which induce apoptosis in cancer cells. We showed that EXOSC4 regulates the stability of *SESN2* mRNA in pancreatic cancer cells; however, we were unable to evaluate the effects of EXOSC4 on *BIK* mRNA because of their low expression levels. Since EXOSC4 is localized in the nucleus of pancreatic cancer cells, EXOSC4 may regulate *BIK* expression via not only mRNA stability but also transcriptional regulation.

BIK functions as a pro-apoptotic tumor suppressor in several human cancers and its expression in cancer is downregulated by chromosomal deletion or transcriptional silencing [44]. BIK is a critical effector in apoptosis that is induced by toxins, cytokines, and virus infection [44]. BIK protein is regulated by the proteasomal machinery [50,51,52]. Moreover, the accumulation of p53 proteins induced the upregulation of *BIK* mRNA in response to several stimuli such as γ radiation, treatment of anti-cancer drugs, and activation of E2F [53,54]. SESN2 represses ROS and plays essential roles in various noxious stimuli including genotoxic and oxidative stress, endoplasmic reticulum (ER) stress, and hypoxia [47]. Although EXOSC4 regulates the expression of *BIK* and *SESN2* mRNA in pancreatic cancer cells, how EXOSC4 recognizes the mRNAs is still unclear. Confirming the direct association of EXOSC4 with target mRNAs is important but challenging because EXOSC4 degrades target mRNAs via the RNA exosome complex and accessory factors. Furthermore, we reported that knockdown of BIK and SESN2 partially rescues growth inhibition caused by EXOSC4 knockdown. We concluded that EXOSC4-mediated downregulation of BIK and SESN2 is required for the proliferation of pancreatic cancer cells. The effect of BIK and SESN2 depletion was only partial presumably because EXOSC4 regulates various genes for cell proliferation. In our next study, we will investigate whether upregulation of other core EXOSC4-regulated genes may affect the inhibition of pancreatic cancer cell growth under EXOSC4 knockdown. GO and GSEA analyses revealed that EXOSC4 might be involved in the lysosome, which is a membrane-bound intracellular organelle that receives macromolecules delivered by endocytosis, phagocytosis, and autophagy for degradation and recycling [55,56]. Lysosomes also play an essential role in the progression of apoptosis [56,57,58,59]. We speculate that EXOSC4-regulated lysosome factors may also regulate cell viability upon EXOSC4 knockdown.

EXOSC4 is one of a part of the barrel structure of RNA exosome and does not have catalytic activity [4]. This suggests that other degradation factors may be involved in the destabilization of *SESN2* mRNA under EXOSC4 depletion. A recent study reported that EXOSC4 knockdown resulted in the downregulation of other RNA exosome components, EXOSC3 and EXOSC9, at the protein level in MDA-MB-231 cells, a breast cancer cell line [49]. EXOSC4 may degrade *SESN2* mRNA levels by regulating EXOSC10 and DIS3 family protein levels in pancreatic cancer cells. In future studies, we will examine whether EXOSC4 is involved in the RNA exosome complex organization or in the incorporation of target mRNAs into the RNA exosome. Moreover, since EXOSC4 is localized in the cytoplasm and mRNAs are usually degraded in the cytoplasm, it is possible that the degradation of *SESN2* mRNA by EXOSC4 is mediated by other RNA degradation mechanisms in the cytoplasm.

The *EXOSC4* gene is located on chromosome 8q24.3. Chromosome 8q24 is the most commonly amplified region across multiple cancer types [60], and amplification and overexpression of this region are associated with poor outcomes in different human tumor types [60,61,62,63]. An array-CGH analysis revealed gain of chromosome 8q in colorectal cancer, and microarray analysis revealed that *EXOSC4* was overexpressed in colorectal cancer [37]. In addition, a genome-wide study revealed that the promoter region of *EXOSC4* was hypomethylated and its expression was upregulated in HCC tumors in comparison with adjacent normal tissue [64]. Further studies showed that EXOSC4 overexpression increased the tumorigenicity of colon cancer cells by promoting cell proliferation and cell invasion [37]. In this study, we also showed that the *EXOSC4* gene was amplified in colorectal cancer, although at a rate of less than 5% (Table S3). Although *EXOSC4* gene amplification was confirmed across multiple tumors, *EXOSC4* gene amplification was associated with poor disease prognosis only in pancreatic cancer patients. These results suggested that EXOSC4 may have important roles, such as tumor progression, particularly in pancreatic cancer and be a good prognostic biomarker and therapeutic target in pancreatic cancer.

In conclusion, we demonstrated that the *EXOSC4* gene is amplified across multiple cancer types and its amplification is associated with poor clinical outcomes in pancreatic cancer. We speculate that EXOSC4 knockdown leads to the reduction in the growth of pancreatic tumor cells by upregulation of *SESN2* and *BIK* mRNA. These findings may provide novel insights into the development of pancreatic cancer treatments. Future studies are needed to address the molecular mechanisms of target mRNA degradation by EXOSC4 in promoting pancreatic cancer development and progression.

## 4. Materials and Methods

### 4.1. Copy Number Alteration and Mutation Analysis

Gene amplification, deletion, and mutation data from 10,967 tumor samples covering 32 tumor types in TCGA Pan-Cancer studies [33] were obtained from the cBioPortal for Cancer Genomics (http://www.cbioportal.org, accessed on 25 December 2021). The copy number for each gene was derived from the GISTIC algorithm, a copy number analysis algorithm, and categorized as copy number level per gene: −2 (deep deletion) indicates a possible homozygous deletion, −1 (shallow deletion) indicates a possible heterozygous deletion, 0 is diploid, 1 (gain) is considered a low-level gain, and 2 (amplification) indicates a high-level amplification. Gene amplification, deep deletion, and mutation frequencies of *EXOSC4* from 32 tumor types were obtained using the “Cancer Types Summary” display from the cBioPortal. Co-expression data of *EXOSC4* were obtained using the “Co-expression” display from the cBioPortal. Gene alteration frequencies of RNA components from Pancreatic Cancer (UTSW, Nat Commun 2015) [38] studies were obtained using the “OncoPrint” display from the cBioPortal. Survival analysis was performed as described previously [34]. Heatmaps were generated using the Morpheus online software suite (https://software.broadinstitute.org/morpheus/, accessed on 25 December 2021).

### 4.2. Gene Expression Analysis

The GEPIA 2 website (http://gepia2.cancer-pku.cn/, accessed on 25 December 2021) was used to examine the gene expressions of *EXOSC4* in pancreatic cancer tissue. The median expression (transcripts per million) of genes in non-tumor and tumor tissues was obtained from the ‘Expression DIY/Box Plot’ section of the GEPIA 2 website. The parameters selected were as follows: |Log2FC| Cutoff, 0.5; *p*-value Cutoff, 0.05; Log Scale, Yes; Jitter Size, 0.4; Matched Normal data; Match TCGA normal; and GTEx data.

### 4.3. Cell Culture

MIA Paca-2 and Hs766T cells (ATCC) were cultured in D-MEM (High Glucose) with L-glutamine and phenol red (044-29765, Wako, Osaka, Japan) supplemented with 10% fetal bovine serum (FBS) and sodium pyruvate (190-14881, Wako). AsPC-1 and SW1990 cells (ATCC) were cultured in RPMI-1640 with L-glutamine and phenol red (189-02025, Wako) supplemented with 10% FBS, sodium pyruvate (190-14881, Wako), HEPES Buffer Solution (345-06681, Wako), and 45 *w*/*v*% D(+)-glucose (079-05511, Wako).

### 4.4. Antibodies and Reagents

Anti-EXOSC4 antibody (HPA024792) was purchased from Sigma-Aldrich, St. Louis, MO, USA. Anti-GAPDH antibody (2955484) was obtained from Merck Millipore (Burlington, MA, USA). HRP-conjugated goat anti-mouse antibody (P0447) and HRP-conjugated goat anti-rabbit antibody (7074S) were obtained from Dako (Denmark), and Cell Signaling Technology Japan (Tokyo, Japan), respectively. ECL-plus was purchased from GE Healthcare (Chicago, IL, USA). We purchased 5, 6-dichloro-1-beta-D-ribofuranosyl benzimidazole (DRB) from Cayman Chemical (Ann Arbor, MI, USA).

### 4.5. Immunohistochemical Analysis

Patient specimens from surgically resected pancreatic cancers were examined using a protocol approved by the Institutional Review Board of the Asahikawa Medical University (# 17002). Protein expression was examined by immunohistochemistry using the anti-EXOSC4 antibody (1:300) on paraffin-embedded sections (4-µm thickness). Antigen retrieval was performed using retrieval buffer (pH 9.0) (Nichirei Biosciences, Tokyo, Japan) and autoclaving at 110 °C for 10 min. Primary antibody reactions were carried out for 1 h at room temperature. Histofine Simple Stain MAX PO (R) (Nichirei Biosciences) was used as instructed by the manufacturer. Visualization of immune reactions was made using 3,3′-diaminobenzidine tetrahydrochloride solution mixed with 0.025% hydrogen peroxide. Nuclei were counterstained with hematoxylin.

### 4.6. Immunofluorescence

Cells cultured on cover slips in 12-well plates were washed twice with PBS plus 0.1% Tween20 and fixed with cold 100% methanol for 5 min at room temperature. The cover slips were washed three times with PBS, permeabilized in 0.1% Triton-X100/PBS for 5 min at 4 °C, and washed three times with PBS. After blocking with 1% BSA/PBS/0.01% Tween20 for 30 min at room temperature, the cells were incubated in 1% BSA/PBS/0.01% Tween20 containing anti-EXOSC4 antibody at 4 °C overnight. After washing three times with PBS plus 0.1% Tween20, 1% BSA/PBS/0.01% Tween20 with Alexa 488-conjugated secondary antibody was added to the cells, and the samples were incubated for 1 h at room temperature. After washing three times with PBS plus 0.1% Tween20, the cells were incubated in DAPI/PBS for 10 min at room temperature. The cover slips were mounted with ProLong gold (P36934, Thermo Fisher Scientific, Waltham, MA, USA), and images were acquired with a ZEISS LSM 980 with Airyscan 2.

### 4.7. RNA Interference

The siRNA duplexes targeting EXOSC3 (s27230, s27231), EXOSC4 (s29112, s29113), EXOSC9 (s10736, s10734), EXOSC10 (s10737, s10738), BIK (s1989), and SESN2 (s38098) were purchased from Ambion. Cells were transfected with RNA duplexes using Lipofectamine RNAiMAX (Thermo Fisher Scientific). Silencer Select negative control siRNA #2 (Ambion) was used as a control.

### 4.8. The qRT-PCR Analysis

Total RNA was isolated using RNAiso Plus (Takara Bio, Shiga, Japan). First-strand cDNA was synthesized with the PrimeScript RT Master Mix (Takara Bio). The qRT-PCR analysis of cDNA was performed on a Thermal Cycler Dice Real Time System (Takara Bio) using TB Green^®^ Premix Ex Taq™ II (Takara Bio). The values were normalized to the levels of GAPDH or HPRT1 mRNA and then fold-change was determined. Primer sequences are listed in Table S19.

### 4.9. Immunoblotting

Cells were lysed for 20 min with lysis buffer (50 mM HEPES pH 7.5, 150 mM KCl, 0.5% NP40, 2 mM EDTA, 1 mM NaF) containing protease inhibitor cocktail (P8340, Sigma-Aldrich). After centrifugation at 13,500× *g* for 20 min at 4 °C, samples were resolved by SDS-PAGE and transferred to PVDF membranes (Immobilon-P, Merck Millipore, Burlington, MA, USA). Membranes were blocked with 5% skimmed milk in TBS plus 0.1% Tween20 for 1 h at 25 °C before immunoblotting using anti-EXOSC4 or anti-GAPDH antibodies and HRP-conjugated secondary antibodies. Visualization was performed using the Immobilon Western chemiluminescent HRP substrate (WBLUF0500, Millipore) and LAS-4000UVmini Luminescent Image Analyzer (FUJIFILM, Tokyo, Japan), according to the manufacturers’ instructions.

### 4.10. Cell Proliferation Assay

Cell viability was determined using the Cell Counting Kit-8 kit (SB056, Dojindo, Kumamoto, Japan) according to the manufacturer’s protocol. Cells were seeded in a 96-well plate at 24 h before transfection. Cells were then transfected with siRNA targeting EXOSC4 or negative control. At 120 h after transfection, 10 µL of CCK-8 solution were added to each well of the plate, and cells were incubated for 90 min. The absorbance at 450 nm was measured using a GloMax Discover Microplate Reader (Promega, Madison, WI, USA).

### 4.11. Apoptosis

Cells were seeded in a 24-well plate at 24 h before transfection and then transfected with siRNA targeting EXOSC4. At 120 h after transfection, cells were washed with PBS and collected into a 15-mL tube. Phosphatidylserine exposure at the cell surface was detected using the MEBCYTO Apoptosis Kit (4700, MBL) according to the manufacturer’s protocol. The percentages of Annexin(+) PI(−) cells and DNA content were measured by flow cytometry (FACSAria Cell Sorter; Becton Dickinson, Franklin Lakes, NJ, USA).

### 4.12. Data Analysis

RNA-seq samples from MIA Paca-2 and AsPC-1 cells transfected with siRNA targeting EXOSC4 were sequenced using the Illumina NovaSeq 6000. Raw reads were mapped to the human reference genome (GRCh38) using HISAT 2.1.0, and gene expression levels were calculated by StringTie 2.1.4. Additional information such as gene symbols and mRNA names were annotated according to GENCODE v32 (GRCh38). Genes with fewer than one fragment per kilobase of exon per million reads mapped were removed. Genes with up- or down-regulated expression by >1.8-fold by EXOSC4 knockdown were selected as differentially expressed genes. Functional analyses were performed using the Enrichr software tool. Gene set enrichment analysis, ridgeplot, and GSEAplot were carried out using R package clusterProfiler [65]. The “MSigDB hallmark all v7.4” gene set was downloaded from the molecular signatures’ database (MSigDB) [66,67]. RNA-seq data were deposited in the DNA Data Bank of Japan Sequence Read Archive (DRA) database (accession number: DRA012188). Microarray data were retrieved from NCBI’s GEO database (GSE43795 [39]). We retrieved the expression values that were log2 transformed and quantile normalized using R package GEOquery [68]. The differential expression analyses were carried out using R package limma and beadarray [69,70].

### 4.13. Statistical Analysis

Statistical analysis was performed using an unpaired two-tailed Student’s *t*-test. A *p*-value < 0.05 was considered to be statistically significant.

## Data Availability

The data presented in this study are available in this article and supplementary material.

## Supplementary Materials

The following are available online at https://www.mdpi.com/article/10.3390/ijms23010496/s1.
