# Supplementary figures and images for: RNA Exosome Component EXOSC4 Amplified in Multiple Cancer Types Is Required for the Cancer Cell Survival
Source: Int J Mol Sci. 2022 Jan 2;23(1):496. doi: 10.3390/ijms23010496 (PMC8745236; doi:10.3390/ijms23010496)

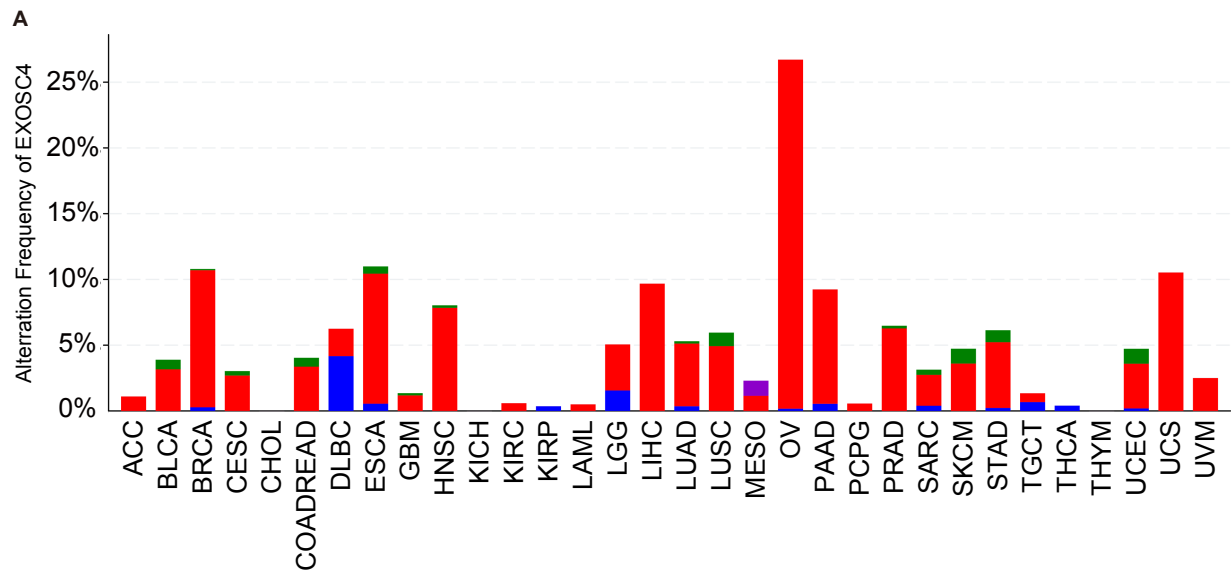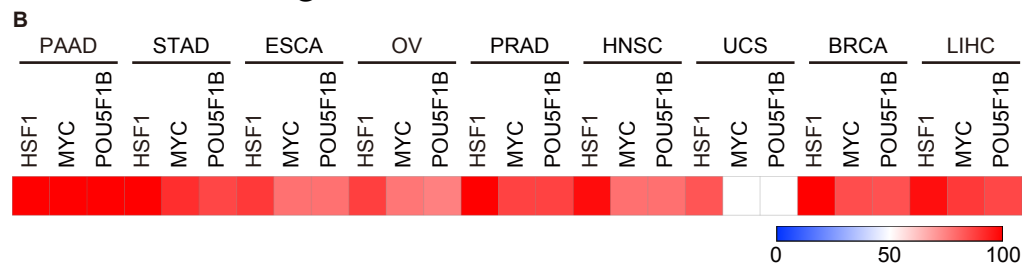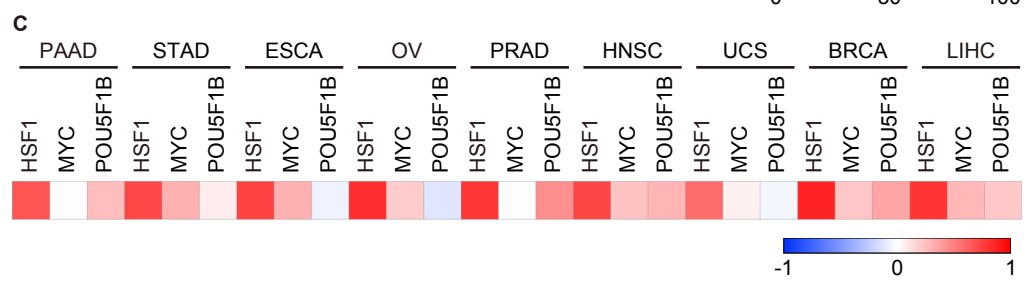

Supplement: Supplementary file 1 [file ijms-23-00496-s001.zip › EXOSC4-figS1.pdf]

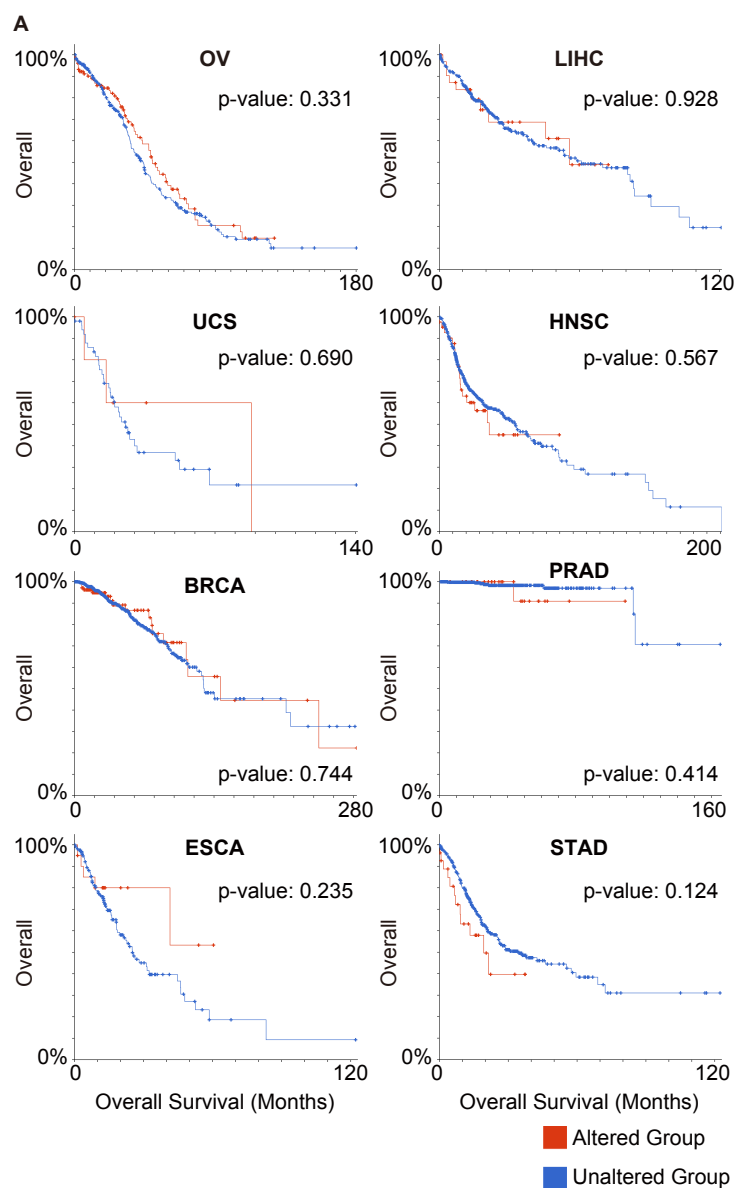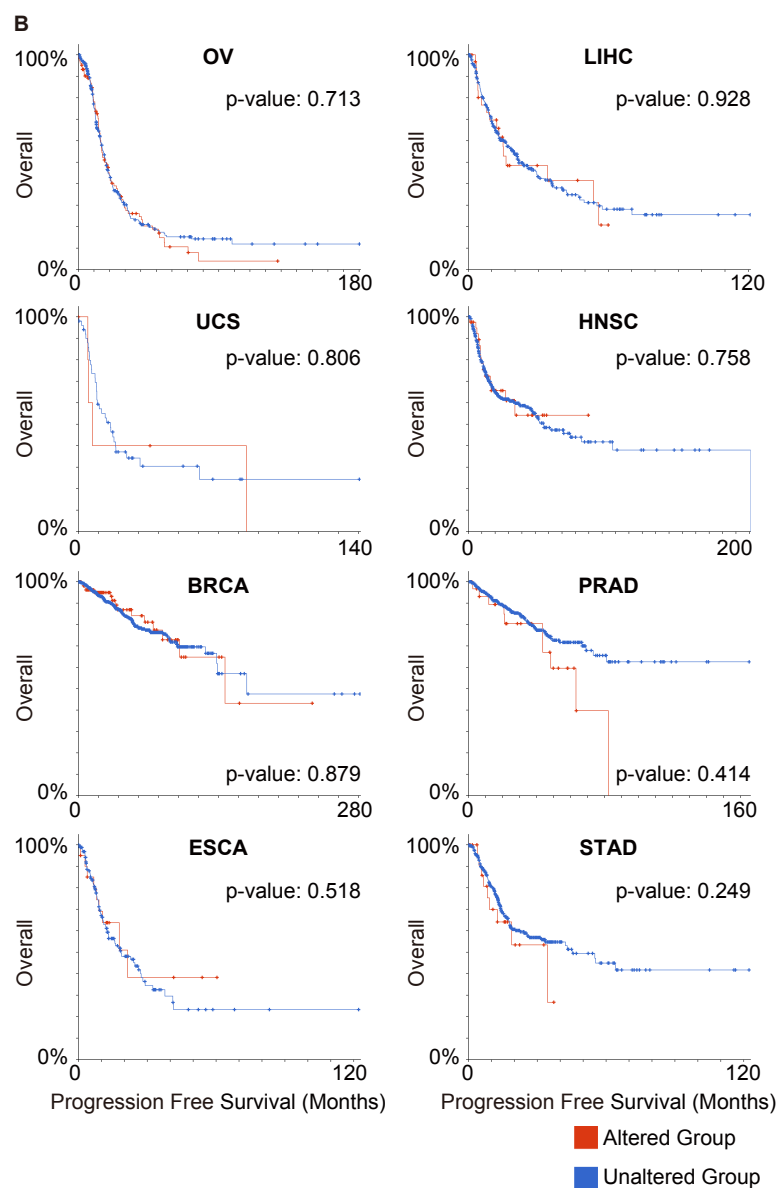

Supplement: Supplementary file 1 [file ijms-23-00496-s001.zip › EXOSC4-figS2.pdf]

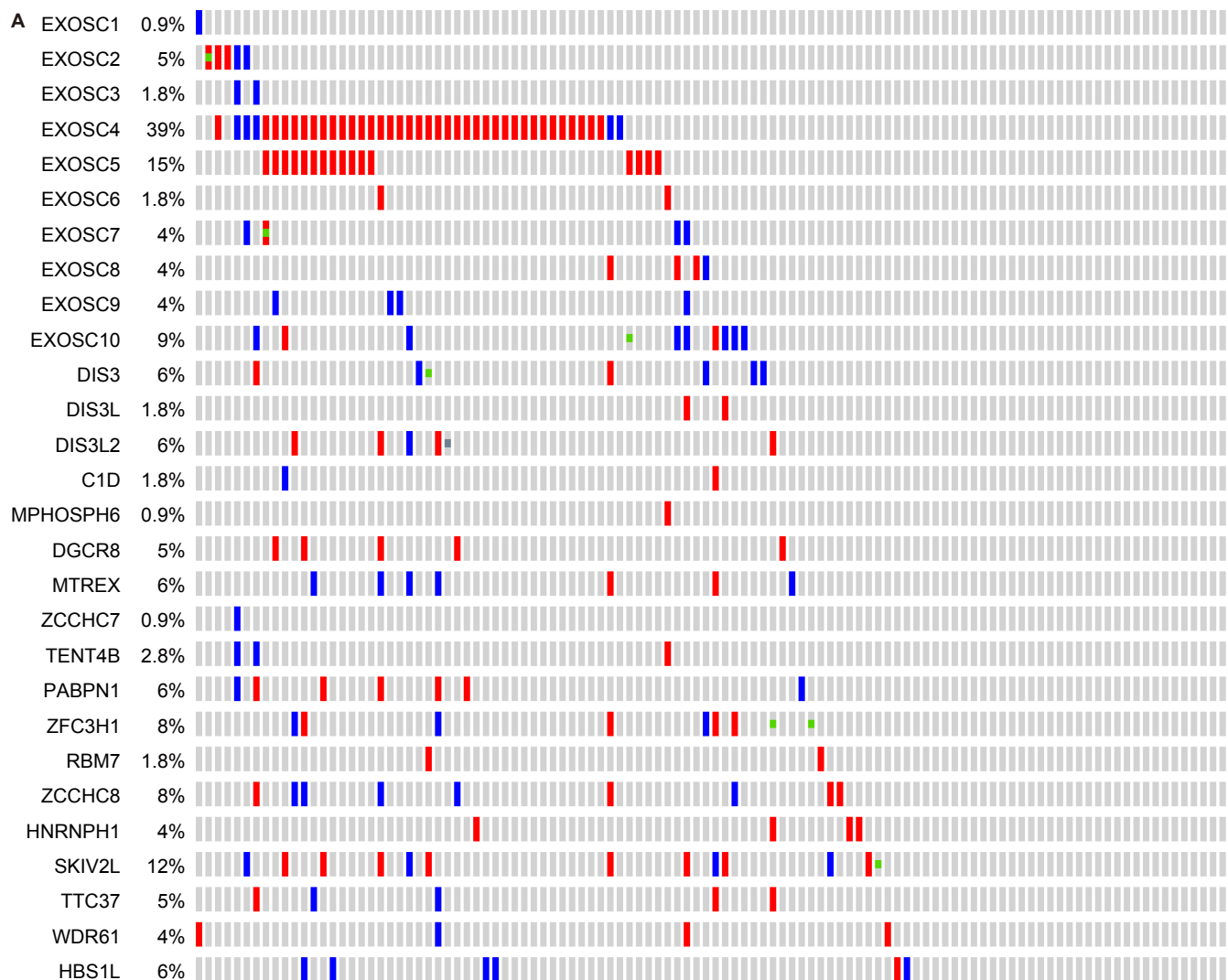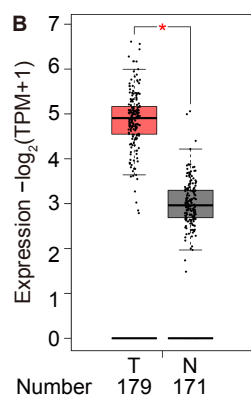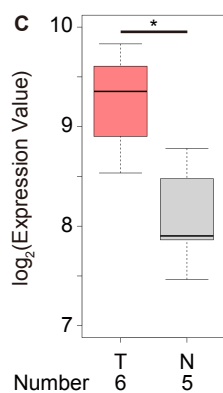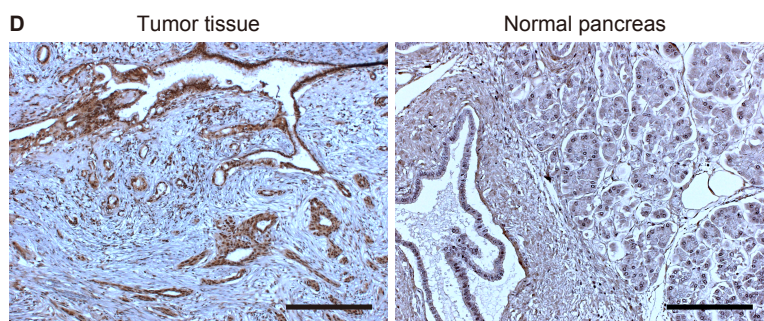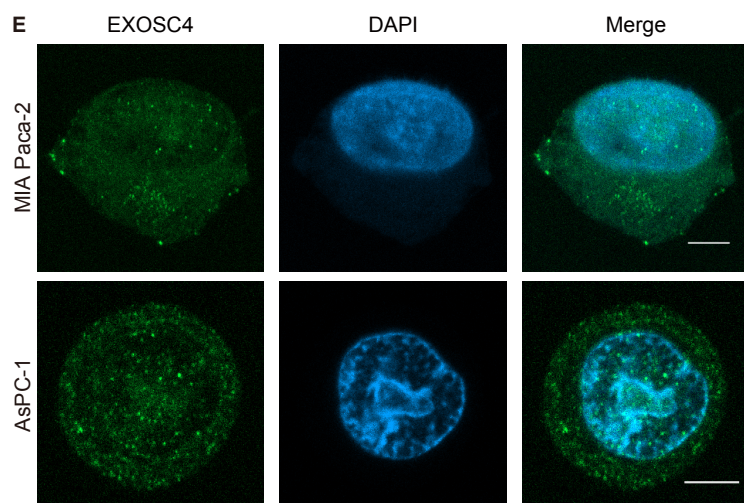

Supplement: Supplementary file 1 [file ijms-23-00496-s001.zip › EXOSC4-figS3.pdf]

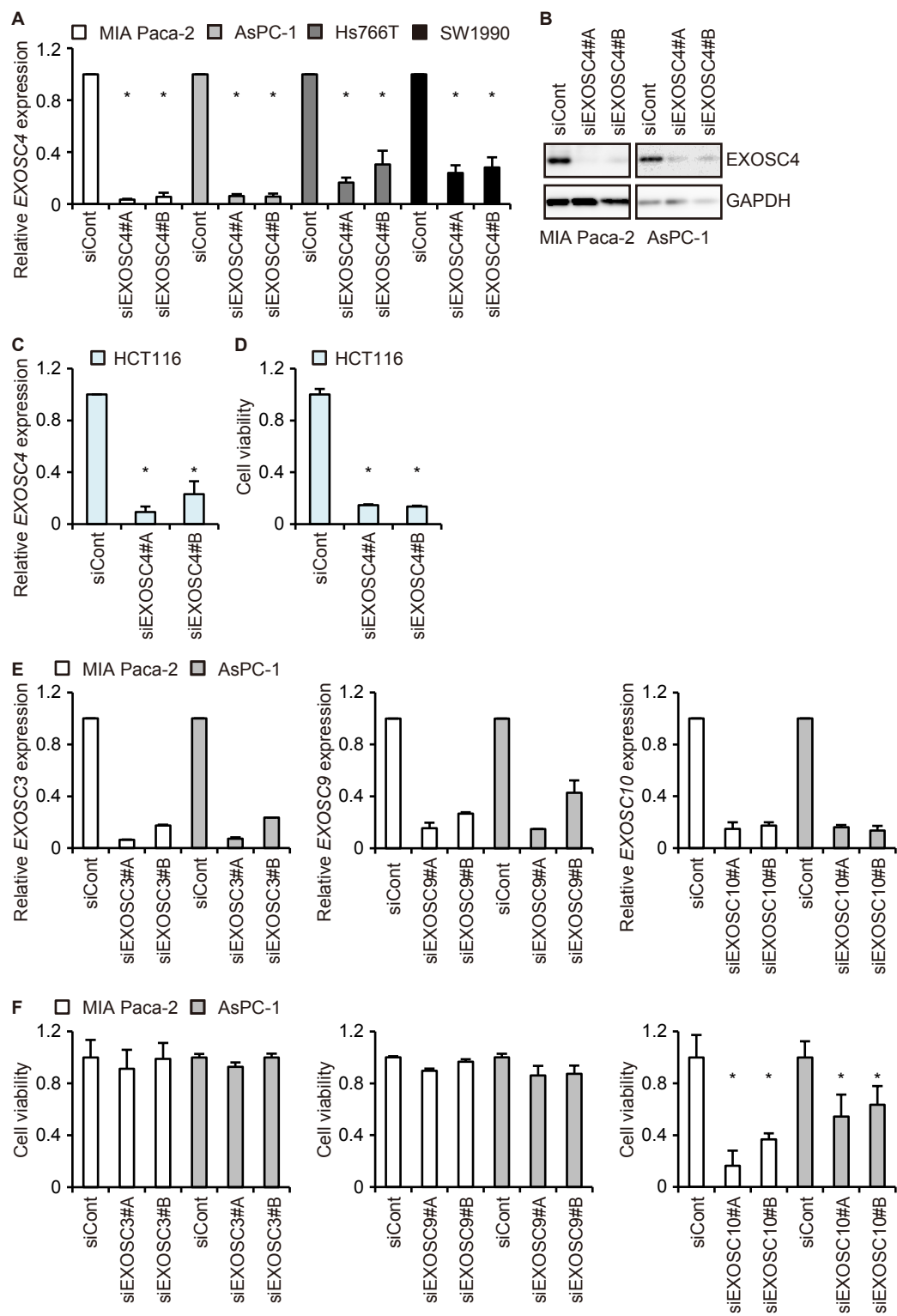

Supplement: Supplementary file 1 [file ijms-23-00496-s001.zip › EXOSC4-figS4.pdf]

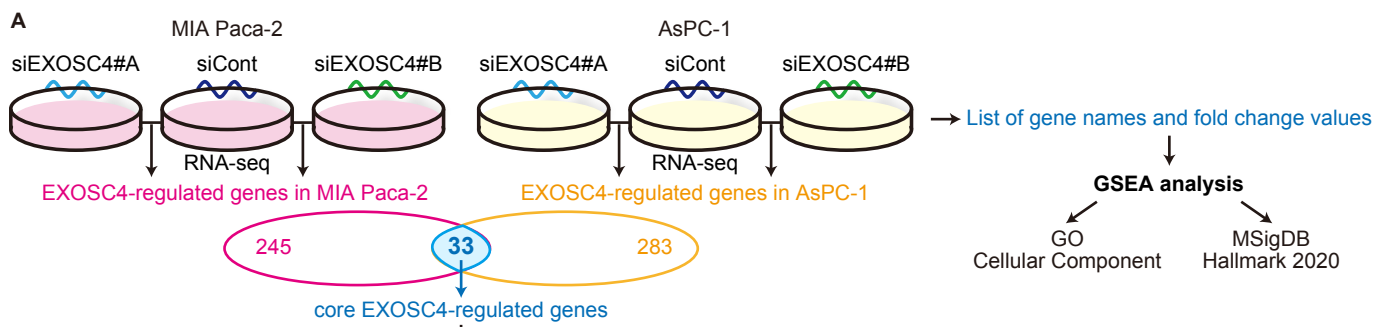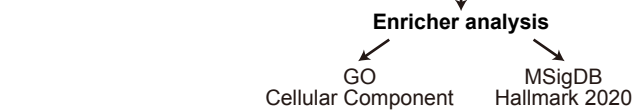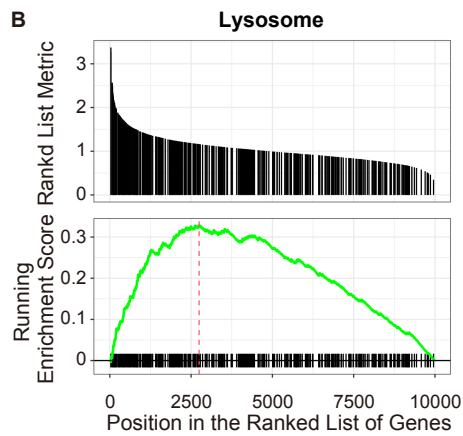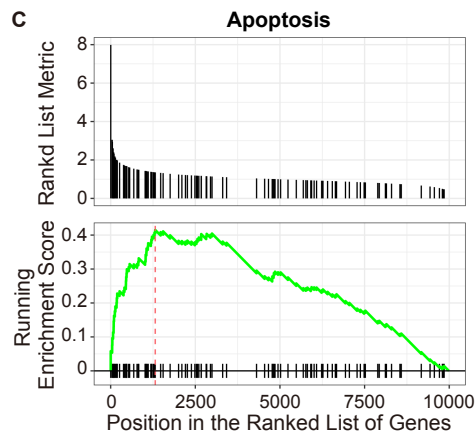

Supplement: Supplementary file 1 [file ijms-23-00496-s001.zip › EXOSC4-figS5.pdf]

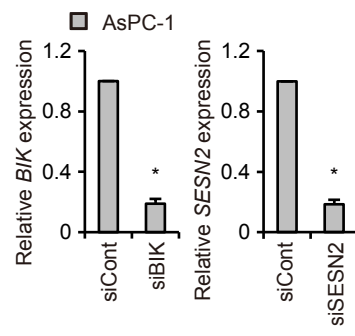

Supplement: Supplementary file 1 [file ijms-23-00496-s001.zip › EXOSC4-figS6.pdf]
